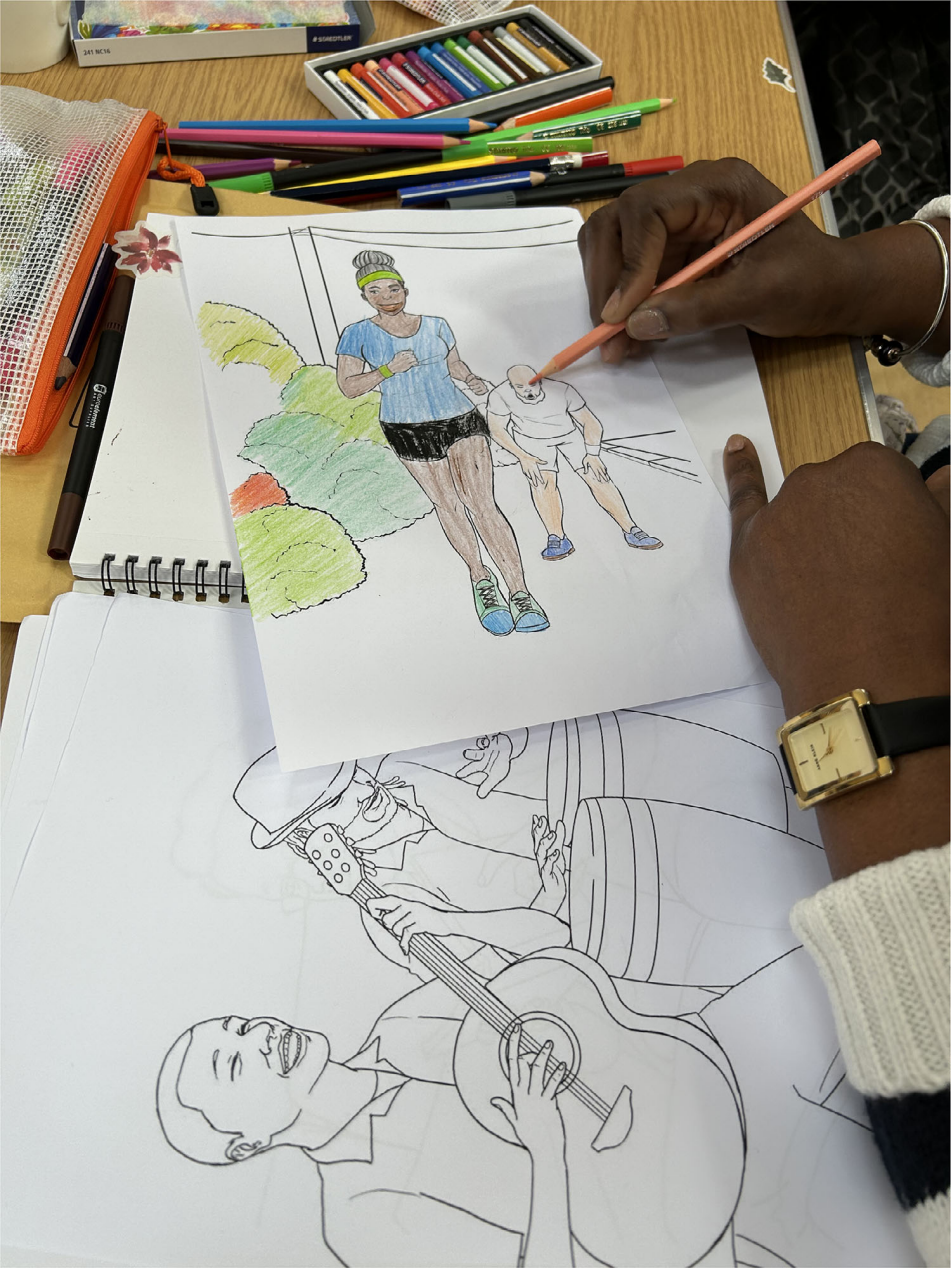# Your Beautiful Brain: Art Workshops for Ethnic Diversity in Dementia Research

**DOI:** 10.1002/alz.093266

**Published:** 2025-01-09

**Authors:** Sarah Bauermeister

**Affiliations:** ^1^ University of Oxford, Oxford, Oxfordshire United Kingdom

## Abstract

**Background:**

Ethnic diversity communities are frequently underrepresented in dementia research. Reasons include lack of identification with dementia research studies, promotional material for dementia prevention and accessibility. The result of this underrepresentation means that new developments for dementia treatments will be not be trialled on these communities. This means that any ethnic differences in dementia or dementia progression will not be taken into account. It is, however, important to understand outreach and education and not to assume that ‘one size fits all’ when it comes to providing information on maintaining a healthy lifestyle for dementia prevention. The aim of Your Beautiful Brain art workshops was to provide a fun activity which was culturally appropriate, for teaching about brain health and dementia prevention. Using art as a medium has a two fold benefit. Firstly, art is fun and secondly, art is a creative activity which is good for the brain, and in the context of the workshops, is also sociable.

**Method:**

The workshops were held and consisted of:

1. A brief talk on healthy lifestyle for a healthy brain

2. Provision of colouring sheets showing Black African and Caribbean community members taking part in healthy activities (e.g., jogging, walking, eating healthy food).

3. Art stationery was provided and included: Art workbook, colouring pencils, pens and pastels, and stickers.

4. A ‘free art’ session was facilitated where participants created any art piece which depicted their understanding of the talk in the morning.

5. Refreshments were provided and the workshops were open to adults aged 50 years and over, both with and without dementia.

**Result:**

6 workshops were held across the United Kingdom, attended by over 50 participants and carers. Feedback from the attendees was that the workshops were very enjoyable and they had learned things about the brain and keeping healthy that they did not know before. Requests to return to provide more workshops were received.

**Conclusion:**

The Your Beautiful Brain art workshops funded by Alzheimer’s Research UK (ARUK) and supported by Dementias Platform UK (DPUK) showed that ethnic communities can be reached through the medium of a fun workshop activity.